# The Evolution of Integrated Interfaces for MEMS Microphones

**DOI:** 10.3390/mi9070323

**Published:** 2018-06-26

**Authors:** Piero Malcovati, Andrea Baschirotto

**Affiliations:** 1 Department of Electrical, Computer, and Biomedical Engineering, University of Pavia, 27100 Pavia, Italy; 2 Department of Physics “G. Occhialini”, University of Milano-Bicocca, 20126 Milano, Italy; andrea.baschirotto@unimib.it

**Keywords:** MEMS microphones, microsensor interface circuits, data converters

## Abstract

Over the last decade, MEMS microphones have become the leading solution for implementing the audio module in most portable devices. One of the main drivers for the success of the MEMS microphone has been the continuous improvement of the corresponding integrated interface circuit performance in terms of both dynamic range and power consumption, which enabled the introduction in mobile devices of additional functionalities, such as Hi-Fi audio recording or voice commands. As a result, MEMS microphone interface circuits evolved from just simple amplification stages to complex mixed-signal circuits, including A/D converters, with ever improving performance. This paper provides an overview of such evolution based on actual design examples, focusing, finally, on the latest cutting-edge solutions.

## 1. Introduction

After the invention of the first microphone in 1876, carbon microphones were introduced in 1878 as key components of early telephone systems. In 1942, ribbon microphones were developed for radio broadcasting. The invention of the self-biased condenser or electret microphones (ECM) in 1962 represented the first significant breakthrough in this field. Indeed, ECMs, ensuring high-sensitivity and wide bandwidth at low cost, have dominated the market for high-volume applications until the last decade, when MEMS microphones started to gain popularity [1].

**Figure 1:**
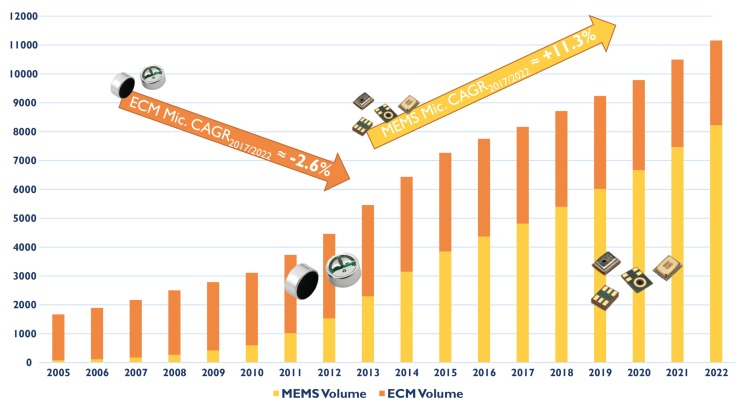
The microphone market in million units since 2005 [3].

The first microphone based on silicon micro-machining (MEMS microphone) was introduced in 1983. Thanks to the use of advanced fabrication technologies, MEMS microphones offer several advantages with respect to ECMs: better performance, smaller size, compatibility with high-temperature automated printed circuit board (PCB) mounting processes, and lower sensitivity to mechanical shocks. Moreover, MEMS microphones can be integrated together with the CMOS electronics on the same chip or, more commonly, within the same package [2], thus reducing area, complexity, and costs, while increasing efficiency, reliability, and performance. As a result, around 2014 MEMS microphones overcame ECMs in terms of sold units, with an annual market size increase of more than 11%, as shown in Figure 1.

MEMS microphones can be realized exploiting different transduction principles, such as piezoresistive, and optical detection. However, more than 80% of the produced MEMS microphones are based on capacitive transduction, since it achieves higher sensitivity, consumes lower power, and is more compatible with batch production. Piezoelectric MEMS microphones are also gaining popularity as an alternative to capacitive devices, since they do not require a biasing voltage, but so far they have not reached the same level of performance and cost effectiveness.

The interface circuit is of paramount importance for MEMS microphones, since it represents one of the most significant competitive advantages with respect to ECMs. Therefore, the development of high-performance interface circuits has been proceeding in parallel with the evolution of MEMS microphones since the very beginning [4,5,6,7,8,9,10,11,12]. The main target in the optimization of these interface circuits is the constant improvement of the audio performances, such as signal-to-noise ratio (SNR), dynamic range (DR), and total harmonic distortion (THD), while maintaining or even reducing the power consumption. This trend is mainly driven by portable applications, in which the audio-related functionalities have been expanding significantly. For example, voice interfaces are becoming pervasive. A growing number of people now talk to their mobile devices, asking them to send e-mails and text messages, to search for directions, or to find information on the internet. These functions require continuous listening, thus introducing severe constraints on the power consumption of the microphone modules. On the other hand, mobile devices nowadays are also used to perform high-fidelity (Hi-Fi) audio/video recording, which require high performance in terms of DR and THD. Such different scenarios are clearly characterized by different performance and power consumption requirements in the microphone module. Different operating modes are required when the same device is re-used in different systems (with different specifications) or when, in the same system, the specifications change depending on the performed function.

In the first case, applications with different DR requirements lead to different component choices, like, for instance, different microphones and/or audio processors. In this situation, the microphone interface circuit has to achieve different performance levels depending on the hardware to which it is connected. In the second case, portable devices supporting voice commands require the audio module to be always active, featuring low DR with low power consumption in stand-by mode (to extend battery life) [13]. However, as soon as an audio input signal is detected, the DR and, hence, the power consumption of the audio module have to be increased to effectively perform the required functions. Then, as soon as the input signal vanishes, the system has to return in stand-by mode. For instance, in always running applications, the bandwidth and DR requirements are typically relaxed (e.g., 4-kHz bandwidth and DR>70 dB), but power consumption has to be extremely low [14,15,16], whereas, in Hi-Fi applications, the required bandwidth is 20 kHz and the DR has to be larger than 90–100 dB, but a relatively high (e.g., around 1 mW) power consumption can be tolerated [17,18,19,20,21,22,23,24].

As a consequence, in the last decade, MEMS microphone interface circuits evolved from just simple amplification stages to complex mixed-signal circuits, including A/D converters, with ever increasing performance.

This paper is organized as follows. Section 2 provides a short overview of MEMS microphones, briefly describing their operating principle. Then, Section 3 discusses the basic principles of the interface circuits for MEMS microphones, illustrating the most important design options and trade-offs, as well as the evolution of both the architecture and the performance over the last decade. This evolution is then analyzed in detail with four actual design examples, which are described in Section 4, Section 5, Section 6 and Section 7, respectively. Finally, in Section 8, we draw some conclusions and discuss future trends.

## 2. Capacitive MEMS Microphones

A microphone is a transducer, which translates a perturbation of the atmospheric pressure, i.e., sound, into an electrical quantity. In a capacitive MEMS microphone, the pressure variation leads to the vibration of a mechanical mass, which, in turn, is transformed into a capacitance variation.

Sound pressure is typically expressed in ${\mathrm{dB}}_{\mathrm{SPL}}$ (sound-pressure-level). A sound pressure of 20 μPa, corresponding to 0 ${\mathrm{dB}}_{\mathrm{SPL}}$, is the auditory threshold (the lowest amplitude of a 1-kHz signal that a human ear can detect). The sound pressure level of a face-to-face conversation ranges between 60 ${\mathrm{dB}}_{\mathrm{SPL}}$ and 70 ${\mathrm{dB}}_{\mathrm{SPL}}$. The sound pressure rises to 94 ${\mathrm{dB}}_{\mathrm{SPL}}$ if the speaker is at a distance of one inch from the listener (or the microphone), which is the case, for example, in mobile phones. Therefore, a sound pressure level of 94 ${\mathrm{dB}}_{\mathrm{SPL}}$, which corresponds to 1 Pa, is used as a reference for acoustic applications. The performance parameters for acoustic systems, such as SNR, are typically specified at 1-Pa and 1-kHz.

**Figure 2:**
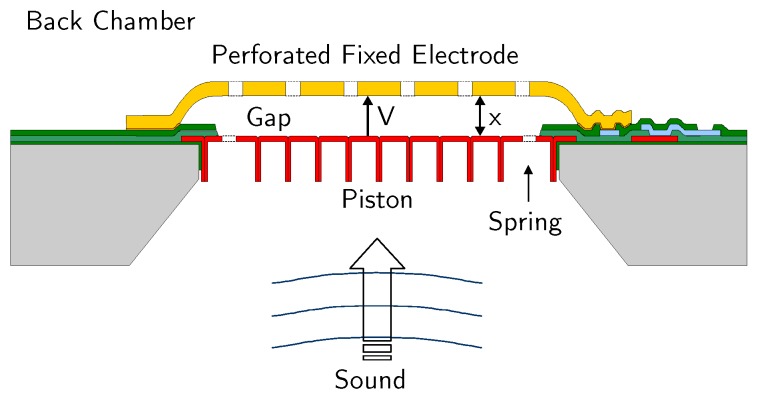
Basic structure and working principle of a MEMS microphone.

A MEMS microphone, whose simplified structure is shown in Figure 2, consists of two conductive plates at a distance *x*. The top plate, in this case, is fixed and cannot move, while the bottom plate is able to vibrate with the sound pressure, producing a variation of *x* (Δx) with respect to its steady-state value ($x_{0}$), proportional to the instantaneous pressure level ($P_{S}$). Different arrangements of the electrodes and fabrication solutions are possible [25,26,27,28,29,30,31], but the basic principle does not change.

The capacitance of a MEMS microphone can then be written as

(1) $$C\left(P_{S}\right)=\frac{\epsilon_{0}A}{x(P_{S})}=\frac{\epsilon_{0}A}{x_{0}+\Delta x\left(P_{S}\right)},$$

where *A* is the area of the smallest capacitor plate and $\epsilon_{0}$ is the vacuum dielectric permittivity.

Denoting with $C_{0}$ the MEMS capacitance in the absence of sound, i.e., when $x=x_{0}$, and assuming linear the relationship between the sound pressure $P_{S}$ and the deformation *x* ($\Delta x=-\kappa \Delta P_{S}$), which is actually true for $\Delta x\ll x_{0}$, we can calculate the output signal (ΔV) as a function of $\Delta P_{S}$. If the MEMS capacitor is initially charged to a fixed voltage $V_{B}$, the charge $Q=C_{0}V_{B}$ remains constant, independently of $P_{S}$. As a consequence, the capacitance variation due to a sound pressure variation $\Delta P_{S}$ leads to a voltage signal (ΔV) given by

(2) $$\Delta V=\frac{Q}{C(P_{S})}-\frac{Q}{C_{0}}=\frac{Q\Delta x}{\epsilon_{0}A}=-\frac{\kappa C_{0}V_{B}\Delta P_{S}}{\epsilon_{0}A}=-\kappa_{V}\Delta P_{S},$$

where $\kappa_{V}$ denotes the voltage sensitivity of the microphone.

According to (2), $\kappa_{V}$ depends on the bias voltage $V_{B}$. Therefore, in order to increase the microphone sensitivity and, hence, the SNR, the value of $V_{B}$ has to be pretty high, typically ranging from 5 V to about 15 V. As a consequence, a charge pump is usually required to generate the desired value of $V_{B}$, starting from the standard CMOS power supply voltage (1.8 V, 2.5 V, or 3.3 V).

**Figure 3:**
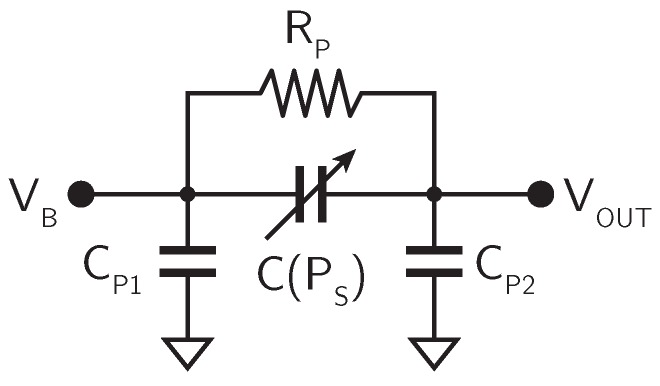
Equivalent circuit of a MEMS microphone.

In practical implementations, a MEMS microphone is not just a capacitor, but some additional parasitic components have to be taken into account. The equivalent circuit of an actual MEMS microphone is shown in Figure 3.

Besides the variable capacitance $C(P_{S})$, the equivalent circuit includes two parasitic capacitances $C_{P1}$ and $C_{P2}$, connected between each plate of the MEMS microphone and the substrate, as well as a parasitic resistance $R_{P}$, connected in parallel to $C(P_{S})$. The value of these parasitic components depends on the specific implementation of the microphone, but typically $C_{P1}$ and $C_{P2}$ are of the order of few pF, while $R_{P}$ is in the GΩ range.

## 3. MEMS Microphone Interface Circuits

**Figure 4:**
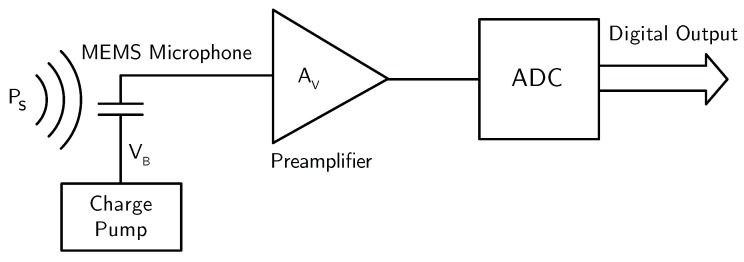
Typical block diagram of the interface circuit for a MEMS microphone.

The interface circuit for a MEMS microphone has to read-out the electrical signal, ΔV, and convert it in the digital domain. Digital output is, indeed, a must for MEMS microphones, in order to gain a competitive advantage over ECMs, in terms of area and cost at system level. Therefore, the interface circuit for a MEMS microphone, whose block diagram is shown in Figure 4, typically consists of a preamplifier followed by an A/D converter (ADC). Moreover, for capacitive MEMS microphones, a charge pump is usually required for generating the microphone bias voltage $V_{B}$. For piezoelectric MEMS microphones, the bias voltage is not required, but, besides this, the interface circuits are basically the same as for capacitive devices.

### 3.1. Preamplifier

**Figure 5:**
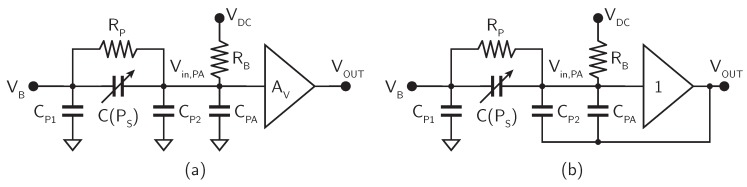
Block diagram of the preamplifier without (**a**) and with (**b**) parasitic capacitance bootstrapping.

The topology and the functionality of the preamplifier in a MEMS microphone interface circuit has to buffer the microphone output voltage, eventually introducing some gain, providing a suitable signal, with low output impedance, to the subsequent ADC, as shown in Figure 5a. In this case, the input impedance of the preamplifier has to be extremely high (larger than 10 GΩ), in order to guarantee that the charge stored on the microphone capacitance is maintained, while providing, at the same time, a suitable DC bias voltage at the preamplifier input node. The biasing network at the preamplifier input is, therefore, very critical and represents typically the most challenging part of the preamplifier design. The solutions usually adopted to implement $R_{B}$ are based on inversely biased diodes or switched networks [32]. Resistor $R_{B}$ introduces a high-pass filter with cut-off frequency $f_{\mathrm{HP}}\approx 1/\left(2\pi R_{B}C_{0}\right)$, which has to be lower than 20 Hz to avoid loss of signal.

The parasitic capacitance at the preamplifier input ($C_{\mathrm{PA}}$) is also particularly important, considering that the output voltage of the microphone ΔV, given by (2), in the presence of parasitic capacitances (both $C_{P2}$ and $C_{\mathrm{PA}}$), is actually attenuated, leading to

(3) $$V_{\mathrm{in},\mathrm{PA}}=\Delta V\frac{C_{0}}{C_{0}+C_{P2}+C_{\mathrm{PA}}}=-\Delta P_{S}\frac{\kappa_{V}C_{0}}{C_{0}+C_{P2}+C_{\mathrm{PA}}}$$

This attenuation can often be quite substantial, thus leading to a degradation of the actual microphone sensitivity and, hence, of the SNR. This problem can be mitigated by bootstrapping $C_{P2}$ and, eventually, also $C_{\mathrm{PA}}$, as shown in Figure 5b. In this case, the voltage across the parasitic capacitances is kept constant, independently of the signal, and, therefore, $V_{\mathrm{in},\mathrm{PA}}\approx \Delta V$. In order to achieve proper bootstrapping, the gain of the preamplifier (or, at least, of the preamplifier first stage) has to be unitary and, hence,

(4) $$V_{\mathrm{OUT}}=-\kappa_{V}\Delta P_{S}$$

Quite often, the overall preamplifier gain is programmable, in order to adapt the microphone output signal range, which can change depending on the used microphone and/or the fabrication tolerances, to the ADC input signal range.

### 3.2. A/D Converter

The large majority of the ADCs for audio applications are realized with sigma-delta (ΣΔ) modulators, in view of their inherent linearity and low power consumption. The main reason that makes ΣΔ modulators particularly suited for audio applications is the relatively small bandwidth of audio signals (B=20Hz,⋯,20kHz), which allows fairly large oversampling ratios, $M=f_{S}/\left(2B\right)$, to be achieved, while maintaining the sampling frequency ($f_{S}$) at acceptable values (few MHz). By trading accuracy with speed, ΣΔ modulators achieve SNR values larger than 60 dB with simple hardware and small area, considering that the SNR of a ΣΔ modulator of order *L* with *N*-bit quantizer and oversampling ratio *M*, is ideally given by [33,34,35]

(5) $$\mathrm{SNR}=\frac{2^{2N}3\left(2L+1\right)M^{2L+1}}{2\pi^{2L}}$$

Following this trend, ΣΔ modulators represent the dominant solution for implementing the ADC also in the interface circuits for MEMS microphones [5,7,9,10,11,12,36,37,38].

Audio ΣΔ modulators can be implemented using either continuous-time (CT) ΣΔ or discrete-time (DT) architectures [33,34]. CT ΣΔ modulators represent the most promising solution for minimizing power consumption, since they require operational amplifiers with lower bandwidth with respect to their DT counterparts for the same SNR. However, they are more sensitive to clock jitter and process variations. The Schreier figure-of-merit [39], defined as

(6) $${\mathrm{FoM}}_{S}=\mathrm{DR}+10\log\frac{B}{P}$$

**Figure 6:**
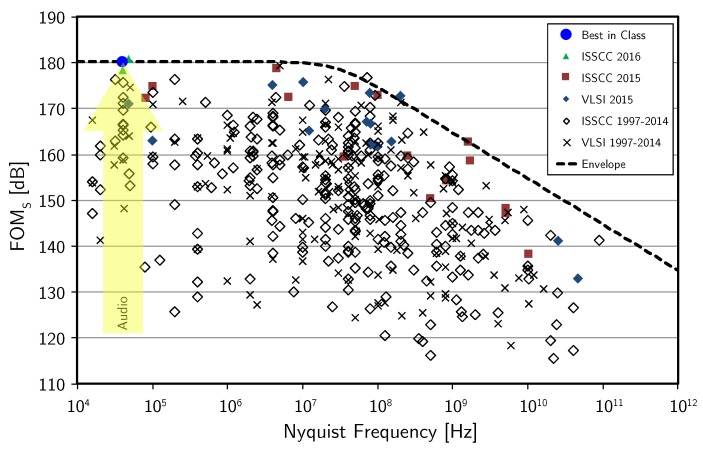
ADC state-of-the-art based on ${\mathrm{FoM}}_{S}$ from [40].

*B* being the bandwidth and *P* the power consumption, is a useful indicator to compare different ADC solutions. Figure 6 shows the values of ${\mathrm{FoM}}_{S}$ of ADCs published in the last 20 years as a function of the Nyquist frequency $F_{N}=2B$.

As expected, the top of class performance in the audio field is achieved with a CT ΣΔ modulator. Moreover, it is possible to verify the trend in the direction of increasing the DR while maintaining or reducing the power consumption, as discussed in Section 1.

To understand this evolution of both the architecture and the performance of the ADCs for MEMS microphones, it is useful to consider four actual design examples, which span from the very first experiments, targeting a DR of the order of 60–70 dB with a power consumption in the mW range, to the latest top-of-class achievements (DR>100 dB with power consumption lower than 1 mW).

## 4. Example 1: Third-Order DT ΣΔ Modulator

**Figure 7:**
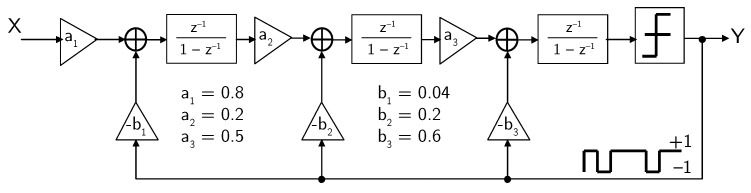
Block diagram of the third-order DT ΣΔ modulator (example 1).

As a first design example, we consider a DT ΣΔ modulator used in one of the very first MEMS microphone interface circuits [7,41]. In this interface circuit, considering the sampling frequency $f_{S}=2.52$ MHz and, hence, the oversampling ratio M=63, according to Equation (5), a third-order (L=3), single-bit (N=1) ΣΔ modulator is sufficient to achieve the required SNR≥60 dB. The block diagram of the third-order DT ΣΔ modulator is shown in Figure 7.

The signal transfer function (STF) and the noise transfer function (NTF) are given by

(7) $$\mathrm{STF}=\frac{0.06z}{\left(z-0.92\right)\left(z^{2}-1.47z+0.55\right)},$$

(8) $$\mathrm{NTF}=\frac{{\left(z-1\right)}^{3}}{\left(z-0.92\right)\left(z^{2}-1.47z+0.55\right)}$$

respectively.

**Figure 8:**
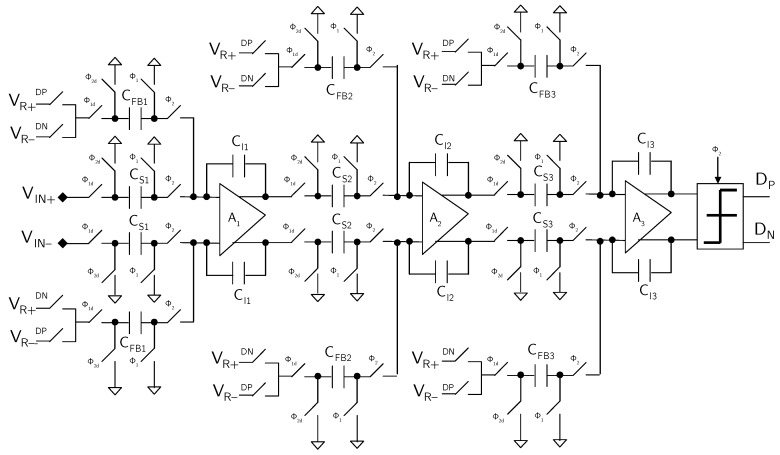
Schematic of the SC implementation of the third-order DT ΣΔ modulator (example 1).

Figure 8 shows the switched-capacitor (SC) implementation of the ΣΔ modulator.

The feedforward and feedback paths are implemented using separate capacitors, thus relaxing the settling requirements of the operational amplifiers. The feedback path contains an extra switch, to select between positive and negative reference voltage ($V_{R+}$ or $V_{R-}$). The first integrator has reduced output swing, but the capacitors are large to keep the kT/C noise low, while the second and third integrator use smaller capacitors, but the output swing is large. Therefore, all the integrators have almost the same settling requirements for the operational amplifiers. Bottom-plate sampling is used in the whole ΣΔ modulator to minimize the distortion due to charge-injection from switches.

The operational amplifiers used for the integrators are based on a telescopic-cascode topology. The common-mode feedback is realized with an SC network. The comparator used consists of a differential stage with regenerative load, followed by a set–reset flip-flop.

### Experimental Results

The interface circuit has been fabricated using a 0.35-μm CMOS technology with four metal and two polysilicon layers. The circuit consumes 210 μA for the analog section and 90 μA for the logic, respectively, leading to an overall power consumption of 1.0 mW with a sampling frequency of 2.52 MHz and a power supply voltage of 3.3 V. The chip area is 3.15 ${\mathrm{mm}}^{2}$ (1930 μm × 1630 μm), including pads.

**Figure 9:**
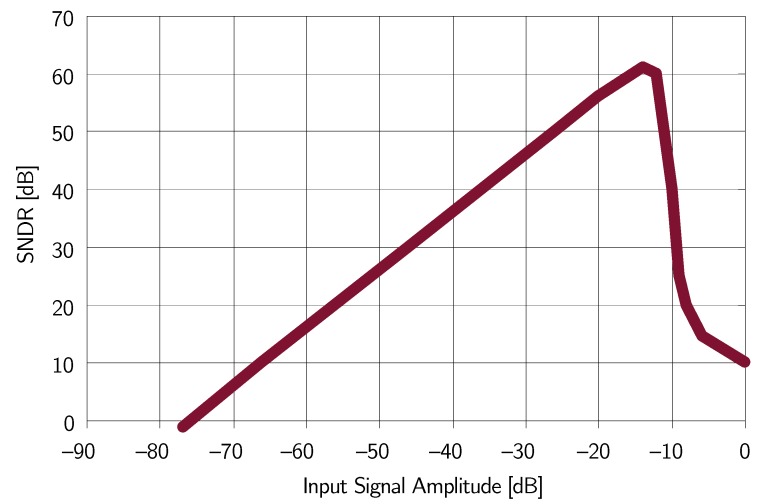
Measured SNDR as a function of the input signal amplitude (example 1).

Figure 9 shows the achieved SNDR as a function of the input signal amplitude with an input signal frequency of 1 kHz. The peak SNDR equal to 61 dB is achieved with an input signal amplitude of −13 ${\mathrm{dB}}_{\mathrm{FS}}$, corresponding to a sound pressure of 104 ${\mathrm{dB}}_{\mathrm{SPL}}$ for the considered MEMS microphone. By considering both noise and distortion contributions, the achieved ENOB is equal to 9.8. The achieved DR is 76 dB.

Finally, Table 1 summarizes the most important measured performances.

## 5. Example 2: Second-Order Multi-Bit DT ΣΔ Modulator

The second design example is a MEMS microphone interface circuit again based on DT ΣΔ modulator [12]. Considering a sampling frequency $f_{S}=2.048$ MHz, with a signal bandwidth B=20 kHz, and hence an oversampling ratio M=51, according to (5), the required SNR≥80 dB and a single-bit output stream can be achieved, for example, with a single-bit quantizer (N=1) and a fourth-order noise shaping (L=4). However, this solution suffers from instability for large input signals, thus requiring watch-dog circuits in order to guarantee saturation recovery. Moreover, at least four operational amplifiers have to be used to design the loop filter.

Another possible solution is to use a 2-2 multi-stage noise shaping (MASH) ΣΔ modulator [33,34] to achieve the required SNR, while overcoming instability issues. However, this solution does not provide a single-bit output stream because of the additional digital filter required to combine the outputs of the cascaded modulators, and suffers from quantization noise leakage problems, due to mismatches between the analog integrators and the digital filter. Moreover, it still requires four operational amplifiers.

**Figure 10:**
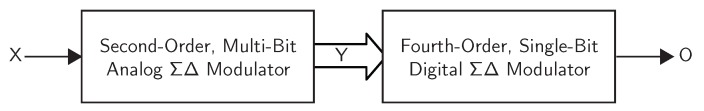
Block diagram of the ΣΔ modulator (example 2).

According to (5), the required SNR is also obtained with L=2 and 3<N<4 (e.g., 12-level quantizer). This solution can be easily designed to be stable even for a large input signal and requires only two operational amplifiers to implement the loop filter. Moreover, multi-bit feedback alleviates the slew-rate requirements of the operational amplifiers. However, this solution does not provide fourth-order noise shaping nor single-bit output stream. These drawbacks can be solved by connecting at the output of the multi-bit, second-order, analog ΣΔ modulator a single-bit, fourth-order, digital ΣΔ modulator, operated at the same sampling frequency $f_{S}$, which truncates the multi-bit output down to a single bit and shapes the resulting truncation error with a fourth-order transfer function. The digital, fourth-order ΣΔ modulator is less critical than its analog counterpart, since it can be easily verified under any operating conditions, and, by using sufficiently large word-length in the integrators and a suitable noise transfer function, instability can be avoided. This solution, whose block diagram is shown in Figure 10, is very promising to achieve the specifications of power consumption and resolution of the system. In order to verify the achievable performance with the used ΣΔ modulator architecture and derive the specifications for the building blocks, behavioral simulations, including most of the non-idealities (kT/C noise, jitter, operational amplifier noise, gain, bandwidth and slew rate), have been performed using a dedicated toolbox [35]. The achieved SNR is 82.4 dB, which corresponds to an effective number of bits (ENOB) of 13.4.

**Figure 11:**
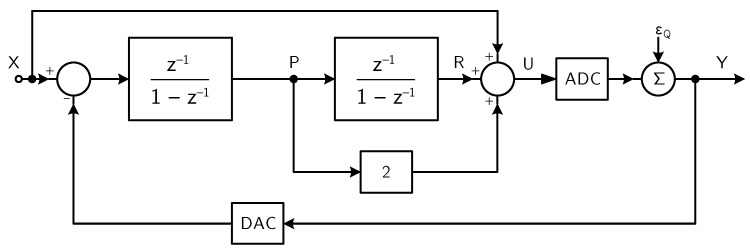
Block diagram of the DT analog second-order ΣΔ modulator (example 2).

Several solutions are available in literature to obtain a DT analog second-order ΣΔ modulator [39]. Among them, the second-order ΣΔ modulator architecture, whose block diagram is shown in Figure 11 [42], is particularly suited for the considered application, since, thanks to the feedforward paths from the input of the integrators to the input of the quantizer, the output of the integrators consists of quantization noise only, thus allowing low-performance (and hence low-power) operational amplifiers to be used.

The analog ΣΔ modulator consists of two integrators, one adder, a flash ADC, and a multi-bit digital-to-analog converter (DAC). The circuit features STF=1, and

(9) $$\mathrm{NTF}={\left(1-z^{-1}\right)}^{2},$$

with second-order noise shaping. Both the integrator outputs consist of quantization noise only, whose maximum amplitude is equal to $V_{\mathrm{ref}}/\left(k+1\right)$, where $V_{\mathrm{ref}}$ is the reference voltage (i.e., the full scale value) and $k=2^{N}$ is the number of levels in the quantizer.

**Figure 12:**
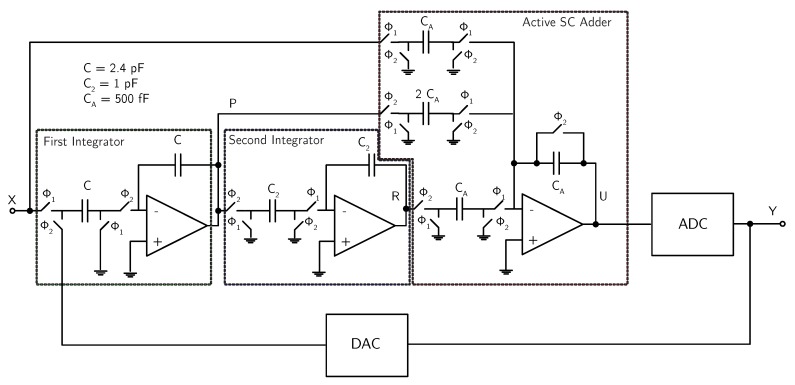
Schematic of the SC implementation of the DT analog second-order ΣΔ modulator (example 2).

Figure 12 shows the SC implementation of the DT analog second-order ΣΔ modulator. The circuit is actually fully-differential, although, for simplicity, Figure 12 shows a single-ended version. An active block has been used to implement the adder before the quantizer, in order to reduce the capacitive load for the two integrators, thus reducing the power consumption. This solution requires an additional operational amplifier but, thanks to the reduced capacitive load, it consumes less power anyway than a solution based on a passive adder.

The operational amplifiers used for the integrators and the adder are based on a folded-cascode topology. The common-mode feedback is realized with an SC network.

The quantizer (flash ADC) consists of k=11 comparators, thus leading to a 12-level output code. The comparator used in the flash ADC consists of a pre-amplifier followed by a clock-driven regenerative latch. The fully-differential comparison between the input signals and the threshold voltages is performed before the pre-amplification stage by an SC network.

The DAC is realized by splitting the input capacitance *C* of the first integrator into 12 identical parts, which are alternately connected to $V_{\mathrm{ref},p}$, $V_{\mathrm{ref},n}$ or $V_{\mathrm{agnd}}$, according to the quantizer output.

**Figure 13:**
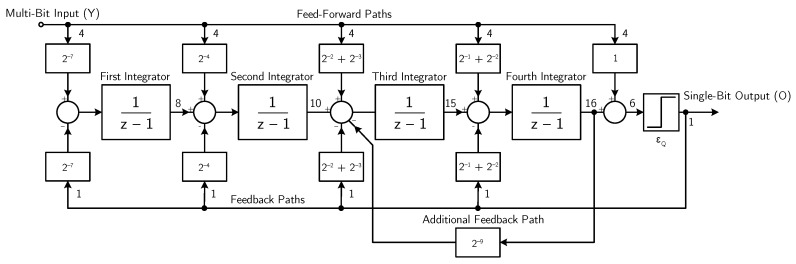
Block diagram of the fourth-order, digital ΣΔ modulator (example 2).

The block diagram of the DT digital fourth-order, single-bit ΣΔ modulator is shown in Figure 13. Denoting with *Y* and $\epsilon_{Q}$ the modulator input and the quantization noise, respectively, the modulator output signal *O* is given by

(10) $$O\left(z\right)=Y\left(z\right)+\epsilon_{Q}\left(z\right)\frac{{\left(z-1\right)}^{2}\left(z^{2}-1.99z+0.99\right)}{\left(z^{2}-1.079z+0.3014\right)\left(z^{2}-1.794z+0.8294\right)}$$

thus leading to a unitary STF in the audio band and an NTF with fourth-order noise shaping. The coefficients of the ΣΔ modulator are implemented as the sum of no more than two terms, each expressed as a power of 2, thus avoiding the use of multipliers.

The word-length in the internal registers is 8 bits for the first integrator, 10 bits for the second integrator, 15 bits for the third integrator, 16 bits for the fourth integrator, and 6 bits for the final adder, in order to avoid saturation and truncation, under any operating conditions.

### Experimental Results

The interface circuit has been fabricated using a 0.35-μm CMOS technology with four metal and two polysilicon layers. The circuit consumes 215 μA for the analog section and 95 μA for the digital section, respectively, leading to an overall power consumption of 1.0 mW with a clock frequency of 2.048 MHz and a power supply voltage of 3.3 V. The chip area is 3 ${\mathrm{mm}}^{2}$ (1755 μm × 1705 μm), including pads. The full-scale input signal amplitude is equal to the DAC reference voltage ($V_{\mathrm{ref}}=V_{\mathrm{ref},p}-V_{\mathrm{ref},n}$), which has been set to ±400 mV, i.e., $V_{\mathrm{in}}=800$ mV peak-to-peak, which, for the considered MEMS microphone, corresponds to about 106 ${\mathrm{dB}}_{\mathrm{SPL}}$.

**Figure 14:**
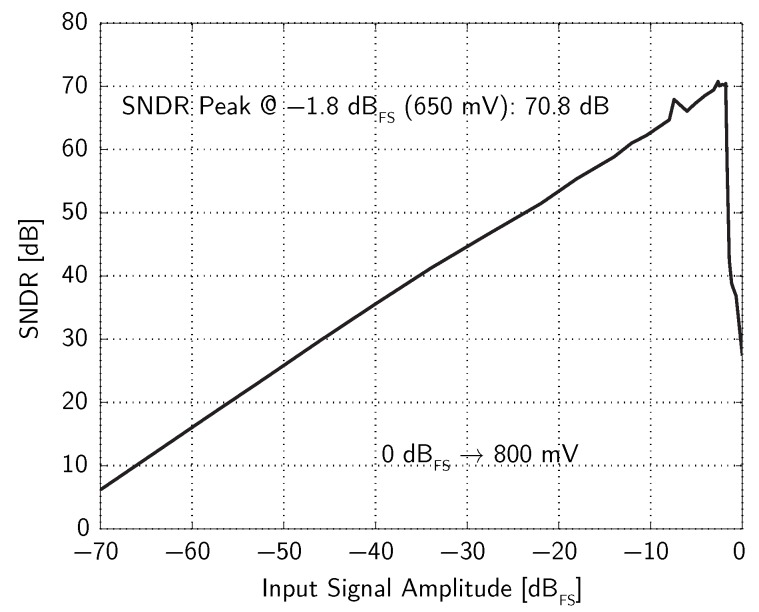
Measured SNDR as a function of the input signal amplitude (example 2).

Figure 14 shows the achieved SNDR as a function of the input signal amplitude with an input signal frequency of 1 kHz. The peak SNDR is equal to 71 dB. By considering both noise and distortion contributions, the achieved ENOB is equal to 11.5. The achieved DR is 77 dB. The use of a feedforward path in the analog, second-order ΣΔ modulator allows the peak SNDR to be achieved for an input signal amplitude as large as −1.8 ${\mathrm{dB}}_{\mathrm{FS}}$.

Finally, Table 2 summarizes the most important measured performances.

## 6. Example 3: Fourth-Order MASH DT ΣΔ Modulator

The third design example belongs to the new generation of MEMS microphone interface circuits. This interface circuit is based on a reconfigurable MASH 2-2 DT ΣΔ modulator, which can efficiently target different functions and/or applications, as discussed in Section 1 [22,24]. The reconfigurable DT ΣΔ modulator can operate in different modes depending on the target function or application. In particular, it is possible to select the ΣΔ modulatror order (second or fourth), the sampling frequency (768 kHz, 2.4 MHz, or 3.6 MHz), the signal bandwidth (4 kHz or 20 kHz), and the bias current level (50%, 75%, or 100% of the nominal value). Among the several resulting operating modes, the three most common ones are:

- Low-Power (LP) mode (second order, $f_{S}=768$ kHz, 4-kHz bandwidth, 50% bias current level);
- Standard (ST) mode (fourth order, $f_{S}=2.4$ MHz, 20-kHz bandwidth, 75% bias current level);
- High-Resolution (HR) mode (fourth order, $f_{S}=3.6$ MHz, 20-kHz bandwidth, 100% bias current level).

**Figure 15:**
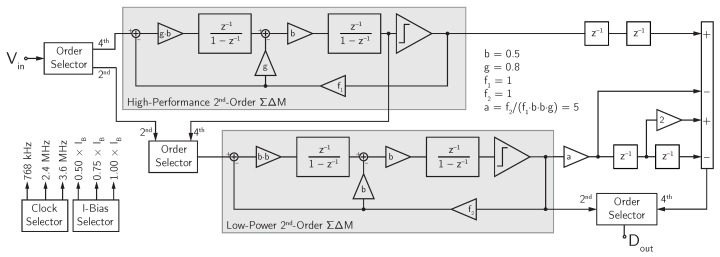
Block diagram of the reconfigurable DT MASH ΣΔ modulator (example 3).

The block diagram of the ΣΔ modulator is shown in Figure 15. It consists of two cascaded second-order stages and a digital recombination filter. The MASH topology has been selected for several reasons. Firstly, it can be made unconditionally stable for input signals bounded within the full-scale, value independently of the operating mode. Moreover, in the presence of accidental signal overload beyond the full-scale value, it guarantees fast recovery. The inherent stability feature allows the SNR to be maintained close to the ideal value given by Equation (5).

With three selectors, it is possible to reconfigure the ΣΔ modulator in a fourth-order or in a second-order topology. When the fourth-order topology is selected, both stages are active, the input is applied to the first stage, the output of the second integrator of the first stage is fed into the second stage, and the multi-bit output is read after the digital recombination network, which merges the bitstreams produced by the two stages. On the other hand, when the second-order topology is selected, only the second stage is active (while the first stage is turned-off), and the input is applied directly to the second stage from which the single-bit output is read.

**Figure 16:**
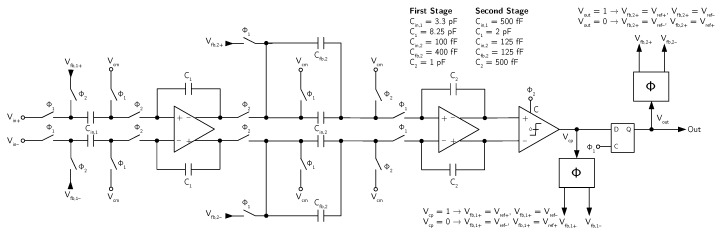
Schematic of the SC implementation of a single stage of the reconfigurable DT MASH ΣΔ modulator (example 3).

The first and the second stages of the DT MASH ΣΔ modulator structure are topologically identical. The fully-differential SC implementation of each second-order stage is shown in Figure 16.

In each second-order ΣΔ modulator stage of the MASH structure, the coefficients are optimized to ensure that the integrator output swing remains within the allowed range under any operating conditions. The coefficients of the digital recombination filter have, then, been set accordingly, in order to properly cancel the first-stage quantization noise from the global ΣΔ modulator output in the operating modes featuring fourth-order noise shaping.

The noise requirements of the second stage are relaxed with respect to the first stage both with fourth-order noise shaping (when the second-stage requirements are reduced by the first-stage gain) and with second-order noise shaping (when lower target specification are required). The softened noise requirements for the second stage are exploited for reducing the capacitance values and the bias current with respect to the first stage. In the same way, inside each stage, the second integrator is designed with lower noise performance (i.e., lower capacitance values and lower bias current) with respect to the first integrator.

### Experimental Results

The reconfigurable MASH SC ΣΔ modulator has been fabricated in a 0.18-μm CMOS process. The chip area is 0.5×0.8 ${\mathrm{mm}}^{2}$, including the ΣΔ modulator, the reference buffers, and an LDO regulator to stabilize the power supply voltage. The reference voltages $V_{\mathrm{ref}+}$ and $V_{\mathrm{ref}-}$ are ±500 mV around the common mode voltage $V_{\mathrm{cm}}=850$ mV (i. e. the ΣΔ modulator full-scale input signal is 2 $V_{\mathrm{pp},\mathrm{diff}}$). These reference voltages are constant independently of the operating mode (they are actually produced by a bandgap reference circuit shared with other blocks in the complete audio module).

**Figure 17:**
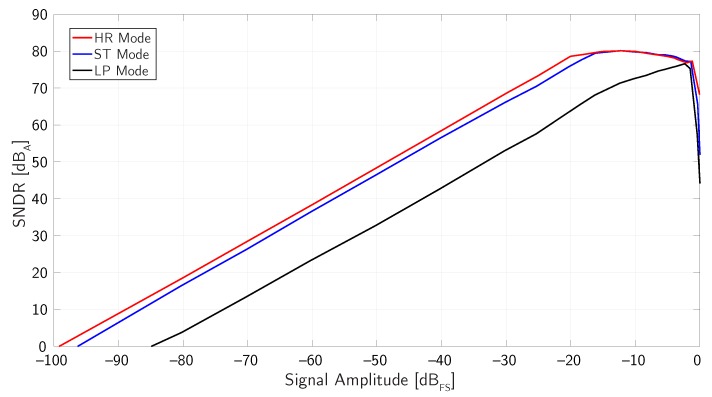
Measured SNDR as a function of the input signal amplitude in the three main operating modes (example 3).

Figure 17 shows the measured SNDR of the ΣΔ modulator as a function of the input signal amplitude at 1 kHz in the three main modes of operation (HR, ST, and LP).

The circuit achieves a DR of 99 dB in HR mode, 96 dB in ST mode, and 85 dB in LP mode. The peak SNDR is limited in all operating modes to about 80 dB by the harmonic distortion of the signal source available for the measurements (in the considered application, the SNDR for sound pressures larger than 100 ${\mathrm{dB}}_{\mathrm{SPL}}$ is anyway limited to about 75 dB by the harmonic distortion of the microphone).

The achieved DR and power consumption of the reconfigurable ΣΔ modulator for all the available operating modes are reported in Table 3, demonstrating the flexibility of the device.

Finally, Table 4 summarizes the the most important measured performances.

## 7. Example 4: Third-Order CT ΣΔ Modulator

**Figure 18:**
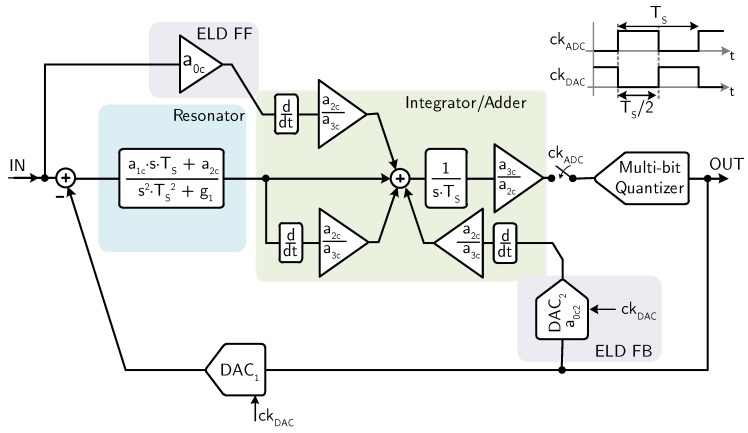
Block diagram of the third-order CT ΣΔ modulator (example 4).

The last example, one of the top-of-class interface circuits for MEMS microphones, is based on a third-order, multi-bit CT ΣΔ modulator [23]. The block diagram of the ΣΔ modulator is illustrated in Figure 18.

The loop filter consists of a resonator (second-order transfer function) followed by an integrator. A local feedback DAC around the quantizer (${\mathrm{DAC}}_{2}$) and a dedicated feedforward path are used for compensating the excess loop delay (ELD). The feedforward paths of the loop filter and the local ELD feedback are differentiated and added at the input of the integrator, in order to avoid an active adder at the input of the quantizer. The multi-bit quantizer drives a 15-level DAC (${\mathrm{DAC}}_{1}$) with dynamic element matching (DEM) to close the main feedback loop of the CT ΣΔ modulator.

**Figure 19:**
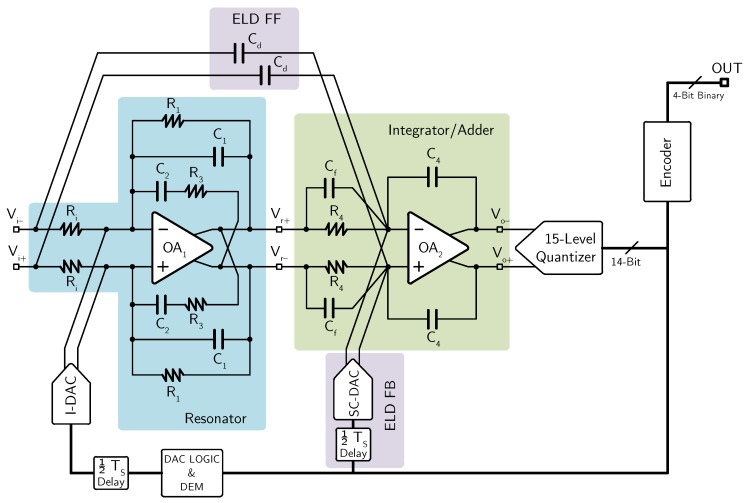
Schematic of the active-RC implementation of the third-order CT ΣΔ modulator (example 4).

The schematic of the active-RC implementation of the CT ΣΔ modulator is shown in Figure 19.

The resonator is implemented using a single operational amplifier and no active adder is used at the input of the quantizer, thus requiring only two operational amplifiers for implementing the third-order loop-filter transfer function. The local feedback DAC for ELD compensation is implemented with an SC structure, whereas the main feedback DAC is realized with a three-level (−1, 0, +1) current-steering topology, which guarantees minimum noise for small input signals. Indeed, with the three-level topology, the unused DAC current sources are not connected to the resonator input and, hence, they do not contribute to the CT ΣΔ modulator noise. The multi-bit quantizer is realized with 14 identical differential comparators and a resistive divider from the analog power supply for generating the threshold voltages.

The values of the passive components used for implementing the CT ΣΔ modulator are summarized in Table 5. The value of $R_{i}$ has been chosen as low as 47 kΩ to fulfill the thermal noise requirements, while $R_{1}$, $R_{3}$, $R_{4}$, $C_{1}$, $C_{2}$, $C_{f}$, and $C_{4}$ are obtained consequently to achieve the desired CT ΣΔ modulator coefficients. Eventually, resistors $R_{i}$ can be removed if the preamplifier is realized with a transconductor which provides directly an output current. Both operational amplifiers are realized with a two-stage, Miller compensated topology in which transistor size and bias current are sized to fulfill the noise requirements (the values in the second operational amplifier are scaled with respect to the first one, since its noise contribution is negligible).

### Experimental Results

The third-order CT ΣΔ modulator has been fabricated using a 0.16-μm CMOS technology. The chip area is 0.21-${\mathrm{mm}}^{2}$.

**Figure 20:**
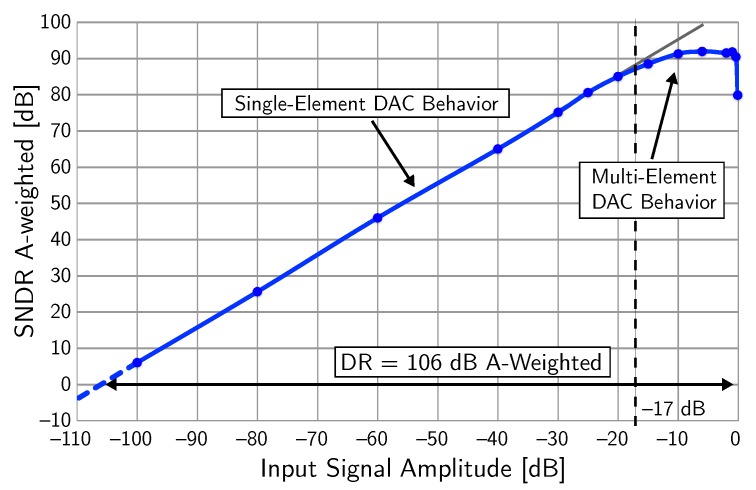
Measured SNDR as a function of the input signal amplitude (example 4).

Figure 20 shows the measured SNDR as a function of the input signal amplitude at 1 kHz. The full-scale input signal (0 ${\mathrm{dB}}_{\mathrm{FS}}$) corresponds to 1 $V_{\mathrm{rms}}$ differential. The achieved DR is 106 dB (A-weighted), corresponding to an ENOB>17 bits, whereas the peak SNDR is 91.3 dB. The change of slope in the SNDR curve for input signal amplitudes larger than −17 ${\mathrm{dB}}_{\mathrm{FS}}$ is due to the increased current-steering DAC noise when more than one three-level DAC element is used (acceptable for the microphone application, where the performance for large input signals is limited by the microphone itself).

The analog section of the third-order ΣΔ modulator consumes 350 μW, while the digital blocks (i.e., DEM and thermometer-to-binary converter) consume 40 μW, both from a 1.6-V power supply and during conversion. The achieved value of ${\mathrm{FoM}}_{S}$ is 180 dB, which is among the highest reported for audio ΣΔ modulators. Table 6 summarizes the achieved performance.

## 8. Conclusions

Looking at the performance evolution in the four reported MEMS microphone interface circuit design examples, summarized in Table 7, it appears clearly that in the last decade the trend has been in the direction of increasing the SNDR and the DR, while maintaining the power consumption in the hundreds of μW range, with the goal of reaching Hi-Fi audio quality (DR>100 dB) in portable devices, eventually introducing some reconfigurability to tackle scenarios, such as voice commands, where a power consumption lower than 100 μW is required. This trend, obviously is reflected in a constant increase of ${\mathrm{FoM}}_{S}$.

Further improvements of the audio quality beyond 110-dB DR are not desirable nor necessary, since the physical limitations in the microphone itself (such as Brownian noise) would anyway prevent the exploitation of such performance at system level. Therefore, the next goal in the development of MEMS microphone interface circuits is toward the reduction of the power consumption below 100 μW, while maintaining the DR performance. Indeed, in this direction, there is still a lot of space for improvements, especially by exploiting the intrinsic features of the audio signals to dynamically adapt the power consumption. Voice activity detection, adaptive biasing, and tracking ADCs are some of the topics being investigated to achieve this target.

## Figures and Tables

**Table 1:** Measured performance summary (example 1).

Parameter	Value
Technology	0.35-μm CMOS
Bandwidth (*B*)	20 kHz
Dynamic range (DR)	76 dB
Signal-to-noise and distortion ratio (SNDR)	61 dB
Effective number of bits (ENOB)	9.8
Power supply voltage	3.3 V
ADC power consumption	360 μW
ADC figure of merit (${\mathrm{FoM}}_{S}$)	153 dB
Total power consumption	1 mW

**Table 2:** Measured performance summary (example 2).

Parameter	Value
Technology	0.35-μm CMOS
Bandwidth (*B*)	20 kHz
Dynamic range (DR)	77 dB
Signal-to-noise and distortion ratio (SNDR)	71 dB
Effective number of bits (ENOB)	11.5
Power supply voltage	3.3 V
ADC power consumption	760 μW
ADC figure of merit (${\mathrm{FoM}}_{S}$)	148 dB
Total power consumption	1 mW

**Table 3:** DR and power consumption in the different operating modes (example 3).

$f_{S}$ [MHz]	*B* [kHz]	Second-Order		Fourth-Order	
		Single-Bit Output		Multi-Bit Output	
		DR [dB]	*P* [mW]	DR [dB]	*P* [mW]
0.768	4	**85 (LP)**	0.10	99	0.48
	20	59		97	
2.4	4	95	0.15	98	0.73
	20	77		**96 (ST)**	
3.6	4	96	0.20	102	0.97
	20	85		**99 (HR)**	

**Table 4:** Measured performance summary (example 3).

Parameter	HR Mode	ST Mode	LP Mode
Bandwidth (*B*) [kHz]	20	20	4
Clock frequency ($f_{S}$) [MHz]	3.6	2.4	0.768
Noise-shaping order (*L*)	4th	4th	2nd
Dynamic range (DR) [dB]	99	96	85
ADC power consumption [mW] ^§^	0.97	0.73	0.10
ADC figure of merit (${\mathrm{FoM}}_{S}$) [dB]	172	170	161
Total power consumption [mW] ^§^	1.33	1.01	0.18
Signal-to-noise and distortion ratio (SNDR) [dB] *	80		
Power supply voltage [V]	1.7–3.6		
Technology	0.18-μm CMOS		

* Limited by the harmonic distortion of the available signal source; ^§^ Measured with a power supply voltage equal to 1.8 V.

**Table 5:** Passive component values in the third-order ΣΔ modulator (example 4).

Resistor	Value	Capacitor	Value
$R_{i}$	47 kΩ	$C_{1}$	18.5 pF
$R_{1}$	5.7 MΩ	$C_{2}$	18.7 pF
$R_{3}$	57 kΩ	$C_{f}$	2.1 pF
$R_{4}$	1 MΩ	$C_{4}$	1 pF

**Table 6:** Measured performance summary (example 4).

Parameter	Value
Technology	0.16-μm CMOS
Bandwidth (*B*)	20 kHz
Dynamic range (DR)	103.1 dB
Dynamic range A-weighted (${\mathrm{DR}}_{A}$)	106 dB
Signal-to-noise and distortion ratio (SNDR)	91.3 dB
Effective number of bits (ENOB)	17
Power supply voltage	1.6 V
ADC power consumption	390 μW
ADC figure of merit (${\mathrm{FoM}}_{S}$)	180 dB

**Table 7:** Evolution of MEMS microphone interface circuits.

Parameter	Example 1	Example 2	Example 3		Example 4
			LP	HR	
Year	2008	2011	2015		2016
Technology	0.35 μm	0.35 μm	0.18 μm		0.16 μm
Bandwidth (*B*) [kHz]	20	20	4	20	20
Noise-shaping order (*L*)	3rd	4th	2th	4nd	3rd
Dynamic range (DR) [dB]	76	77	85	99	103
	$\overset{\;\mathrm{Evolution}\;}{\to }$				
ADC power consumption [mW]	0.36	0.76	0.10	0.97	0.39
ADC figure of merit (${\mathrm{FoM}}_{S}$) [dB]	153	151	161	172	180
	$\overset{\;\mathrm{Evolution}\;}{\to }$				
Power supply voltage [V]	3.3	3.3	1.7–3.6		1.6
